# Roxadustat (FG-4592) Facilitates Recovery From Renal Damage by Ameliorating Mitochondrial Dysfunction Induced by Folic Acid

**DOI:** 10.3389/fphar.2021.788977

**Published:** 2022-02-25

**Authors:** Xue Li, Bo Jiang, Yu Zou, Jie Zhang, Yuan-Yuan Fu, Xiao-Yue Zhai

**Affiliations:** ^1^ Department of Histology and Embryology, Basic Medical College, China Medical University, Shenyang, China; ^2^ Department of Nephrology, Shengjing Hospital of China Medical University, Shenyang, China; ^3^ Department of Vascular Surgery, First Hospital of China Medical University, Shenyang, China; ^4^ Institute of Nephropathology, China Medical University, Shenyang, China

**Keywords:** FG-4592, HIF-1α, repair, mitochondria dysfunction, FA-induced renal damage

## Abstract

Incomplete recovery from acute kidney injury induced by folic acid is a major risk factor for progression to chronic kidney disease. Mitochondrial dysfunction has been considered a crucial contributor to maladaptive repair in acute kidney injury. Treatment with FG-4592, an inhibitor of hypoxia inducible factor prolyl-hydroxylase, is emerging as a new approach to attenuate renal damage; however, the underlying mechanism has not been fully elucidated. The current research demonstrated the protective effect of FG-4592 against renal dysfunction and histopathological damage on the 7th day after FA administration. FG-4592 accelerated tubular repair by promoting tubular cell regeneration, as indicated by increased proliferation of cell nuclear antigen-positive tubular cells, and facilitated structural integrity, as reflected by up-regulation of the epithelial inter-cellular tight junction molecule occludin-1 and the adherens junction molecule E-cadherin. Furthermore, FG-4592 ameliorated tubular functional recovery by restoring the function-related proteins aquaporin1, aquaporin2, and sodium chloride cotransporter. Specifically, FG-4592 pretreatment inhibited hypoxia inducible factor-1α activation on the 7th day after folic acid injection, which ameliorated ultrastructural abnormalities, promoted ATP production, and attenuated excessive reactive oxygen species production both in renal tissue and mitochondria. This was mainly mediated by balancing of mitochondrial dynamics, as indicated by down-regulation of mitochondrial fission 1 and dynamin-related protein 1 as well as up-regulation of mitofusin 1 and optic atrophy 1. Moreover, FG-4592 pretreatment attenuated renal tubular epithelial cell death, kidney inflammation, and subsequent interstitial fibrosis. In vitro, TNF-α-induced HK-2 cells injury could be ameliorated by FG-4592 pretreatment. In summary, our findings support the protective effect of FG-4592 against folic acid-induced mitochondrial dysfunction; therefore, FG-4592 treatment can be used as a useful strategy to facilitate tubular repair and mitigate acute kidney injury progression.

## Introduction

Acute kidney injury (AKI), characterized by renal dysfunction, is involved in failure to maintain important physiological parameters such as volume and electrolyte balance (Kellum et al., 2017). AKI is associated with increased rates of mortality and morbidity (Chan et al., 2020). Ischemia and toxicity are common causes of AKI, which are the major contributors to chronic kidney disease (CKD) and are closely associated with aberrant repair (Humphreys et al., 2016). This relationship highlights how renal recovery from AKI may determine long-term outcomes; however, no effective interventions are currently available to alter the natural course (Bao et al., 2018). Therefore, it is essential to explore the possible mechanisms and seek novel therapeutic options involving maximization of kidney repair to ameliorate AKI prognosis (Lin and Hsu 2020). The regenerative capacity of tubules is limited; in comparison, tubular cells have more regenerative capacity after injury, although the mechanisms are unknown (Soofi et al., 2020). Moreover, maladaptive repair could lead to CKD, which is closely related to cell death and continuous inflammation, eventually leading to increased extracellular matrix accumulation (Gibbs et al., 2018).

Folic acid (FA)-induced AKI is mainly caused by tubular crystal formation and oxidative stress, which leads to epithelial necrosis and inflammation (Gupta et al., 2012). This further exacerbates persistent tissue hypoxia, and the tissue usually cannot fully recover; the condition may easily progress to CKD. Thus, FA-induced AKI model is becoming a valuable tool to explore the mechanisms of maladaptive repair of AKI (Basile et al., 2012; Aparicio-Trejo et al., 2019). In addition, AKI can trigger the degradation of cell-cell tight junction (TJ) and adherens junction (AJ) proteins, including the major transmembrane proteins E-cadherin and zonular occludin-1 (ZO-1). Furthermore, the potential of tubular cells to regenerate is pivotal for reestablishment of tubule integrity (Zhou et al., 2012; Jiang et al., 2016a). Moreover, structural integrity is indispensable for restoration of tubular barrier function, especially the regulation of body fluid volume. Aquaporins (AQPs), which are water channel proteins, are expressed in different segments of the tubule that maintain the normal urine concentration and volume (He and Yang 2019). Moreover, the kidneys play an important role in regulating the balance of electrolytes. For example, sodium chloride cotransporter (NCC), which plays an essential role in ion transport, is mostly expressed in the distal convoluted tubule and drives water reabsorption through active sodium transport (Gamba 2012).

Furthermore, tubular recovery from AKI is associated with a high metabolic demand to perform intense reabsorption processes. Mitochondria are the central energy sources in tubular epithelial cells, and mitochondrial dysfunction has been identified as a critical contributor to abnormal kidney repair (Cheng et al., 2020). Moreover, mitochondria are dynamic organelles that undergo constant fusion and fission, which are balanced to maintain mitochondrial homeostasis under physiological conditions. Excessive mitochondrial fission results in mitochondrial fragmentation, which is found in different models of AKI (Sun et al., 2019). Mitochondrial dysfunction could further impair ATP production and enhance the release of oxides that induce iron-dependent cell death (Battaglia et al., 2020). Moreover, ferroptosis has been reported to be the major cause in FA-induce renal damage, which could be a driver of other pathways of cell death (Diego, Martin-Sanchez et al., 2016; Li et al., 2020). While specific expression of GPX4 can play an important role in anti-oxidative stress, and further inhibit cell ferroptosis (Reichert et al., 2020).

Mitochondrial dysfunction has been found to be the main pathogenic factor in FA-induced renal damage. Restoring the generation of functional mitochondria is essential for cell survival and measures that ameliorate mitochondrial dynamics that could accelerate endogenous regeneration processes, further facilitating recovery from AKI (Reichert et al., 2020). Mitochondrial fusion, primarily driven by mitochondrial fusion proteins such as optic atrophy type 1 (Opa1) on the inner mitochondrial membrane (IMM) and mitofusin 1 (Mfn1) and mitofusin 2 (Mfn2) on the outer mitochondrial membrane (OMM), has been demonstrated to be able to protect against renal damage (Zhan et al., 2013). Mitochondrial fission is mainly regulated by dynamin-related protein 1 (Drp1), a dynamin-related GTPase, which is regulated by anchor protein fission protein 1 (Fis1) (Zhan et al., 2013). Studies have shown that both Opa1 and Mfn2 can maintain the stability of mitochondrial cristae and promote mitochondrial fusion. However, persistent upregulation of Drp1 contributes to mitochondrial fragmentation, which has been found to occur in FA-induced AKI (Aparicio-Trejo et al., 2019). Hypoxia inducible factor-1α (HIF-1α), the transcriptional regulator that responds to hypoxia, has been reported to negatively modulate mitochondrial dynamics by promoting the expression of Drp1 (Chen et al., 2019). In addition, HIF-1α has been reported to inhibit the expression of Mfn2, which directly or indirectly influences mitochondrial function (Martin et al., 2014; Dabrowska et al., 2015).

FG-4592, an inhibitor of prolyl-4-hydroxylases (PHDs), can stabilize the level of HIF-1α through inhibition of PHDs under physiological conditions (Wu et al., 2016). Pretreatment with FG-4592 has been reported to protect against AKI through antiapoptotic effects (Yang et al., 2018). Additionally, in our previous study, FG-4592 pretreatment exerted potential protective effects against FA-induced tubular injury at the acute phase through anti-ferroptosis with up-regulation of HIF-1α, but a decreased level of HIF-1α mRNA was observed, which was probably related to negative feedback (Li et al., 2020). To date, the effect of FG-4952 on FA-induced tubular damage at the repair phase has not been well studied, and the underlying mechanism is still unknown. It has been reported that kidney recovery occurs from the 6th day after FA overdose injection. In this regard, we speculate that the protein level of HIF-1α is down-regulated with time because of the short half-life of the protein and the inhibition of HIF-1α mRNA expression with FG-4592 pretreatment, which may ameliorate mitochondrial dysfunction induced by FA injection. These findings are important because suppressing mitochondrial fission and promoting mitochondrial fusion are promising therapeutic approaches to restore the balance between fission and fusion, which is an attractive strategy for tubular repair and amelioration of AKI prognosis.

## Materials and Methods

### Animals

The protocols were abided by the NIH Policy on Animal Care and Use, and complied with the ethics committee of the China Medical University on Laboratory Animals (protocol no. 2011037). Six- to eight-week-old C57BL/6 male mice were obtained from China Medical University and housed in a specific pathogen-free facility. They were allocated to four groups (six mice for each group): (1) Control group, the mice were intraperitoneally administrated with 0.5 ml of 300 mM NaHCO_3_; (2) Folic acid (FA) group, the mice were intraperitoneally injected with one dose of FA (250 mg/kg) diluted in 300 mM NaHCO_3_; (3) FG-4592 group, the mice were intraperitoneally injected with FG-4592 (10 mg/kg) diluted in DMSO and then PBS to 1 mg/ml; (4) FA + FG-4592 group, the mice were injected with one dose of FG-4592 2 days before FA injection. At day 7 after FA injection, tissue and blood samples were obtained for further test.

### Cell Culture and Treatment

Human proximal tubule epithelial cells (HK-2 cells), purchased from American Type Culture Collection, were cultured in DMEM/F-12 medium supplemented with 10% bovine serum albumin, 100 μg/ml streptomycin, and 100 U/ml penicillin (Invitrogen, Carlsbad CA, United States). Cells were grown to the confluence of 50% and stimulated with TNF-α (50 ng/ml) for 24 h in the presence or not of FG-4592 pretreatment for 24 h.

### Reagents and Antibodies

Rabbit anti-Mfn1 (14739s), rabbit anti-Opa1 (67589s), rabbit anti-Fis1 (86668s), rabbit anti-Drp1 (8570s), rabbit anti-IL-1β (12703s), rabbit anti-CD3 (26582s), rabbit anti-Myeloperoxidase (MPO^+^) (14569T), rabbit anti-F4/80 (30325s), and rabbit anti-collagen I (72026T) antibodies were taken from CST; mouse anti-β-actin (ab8226), rabbit anti-HIF-1α (ab216842), mouse anti-PCNA (ab29), rabbit anti-E-cadherin (ab76319), rabbit anti-NCC (ab203674), rabbit anti-α-SMA (ab5694), mouse anti-vimentin (ab92547), rabbit anti-GPX-4 (ab125066), and rabbit anti-fibronectin (Fn) (ab2413) antibodies were obtained from Abcam; rabbit anti-TNF-α (7B8A11) antibody was purchased from Proteintech; rabbit anti-ZO-1 (PA524716), rabbit anti-AQP1 (101AP), and rabbit anti-AQP2 (201AP) antibodies were obtained from Invitrogen; anti-IL-33 (AF3626) antibody was acquired from R&D Systems. FG-4592 was obtained from Selleck and FA was acquired from Dalian Meilun Biotechnology Co. of China.

### Cell Counting Kit-8 Assay

Cell viability was assayed with the CCK-8 kit (Beyotime, China, C0038). In brief, HK-2 cells were treated with FG-4592 (5–80 µM) for 24 h, or HK-2 cells were treated with TNF-α (50 ng/ml; abs00847, absin, China) for 24 h with and without 24 h pretreatment with FG-4592 (20 µM), then 10 µl CCK-8 solution was added to incubate for 2 h. The absorbance was measured at 450 nm.

### Assays for Renal Function and ROS

In brief, blood was collected from the retro-orbital vein of the mice and serum was acquired by centrifugation at 600 X g for 10 min. BUN was assessed with a BUN test kit (C013-2) and Creatinine was assessed with the Creatinine determination kit (C011-2) from Jiancheng, China, by enzymatic colorimetric methods according to the manufacturer’s instructions. Then the levels of ROS in kidneys were assessed by DCFH-D test kit according to the manufacturer’s instructions (Beyotime, China, S0033) (Shen et al., 2020).

### Tissue ATP Levels

ATP levels were measured using the ATP test kit (Beyotime, China, S0026). Renal tissue was lysed in the lysis solution, then centrifuged at 16,000g for 5 min. Then the ATP levels were assessed by mixing the equal volume of the supernatant and luciferase reagent, and the chemiluminescence was measured.

### Histology

Kidney samples were fixed in 4% paraformaldehyde overnight, dehydrated in an ethanol gradient, cleared in xylene, and embedded in paraffin. Then 3-μm slides were used for hematoxylin and eosin (H&E) staining (Shen et al., 2021), periodic acid-schiff (PAS) staining (Chen et al., 2018), and Masson’s trichrome staining (Fan et al., 2017) to evaluate histopathologic injury. At last, the slides were viewed using Nikon 90i microscope. The H&E sections were used to access degrees of tubular damage from 10 fields in each kidney slide (Brooks et al., 2009).

### Immunofluorescence Staining

The slides were prepared according to the routine procedure, and IF staining was performed as described previously (Li et al., 2020). Kidney samples were antigen retrieved in 10 mM sodium citrate buffer, washed with PBS, and blocked with goat serum. Then, they are incultured with primary monoclonal antibody anti-CD3 (1:200), anti-MPO^+^ (1:200), and anti-collagen I (1:200) antibodies overnight. Subsequently, the slides were incubated with TRITC-conjugated or FITC-conjugated secondary antibodies.

### Immunohistochemical Staining

The sections were performed in accordance with the previous protocals (Li et al., 2020). Briefly, kidney slides were incubated in antigen retrieval buffers, boiled with high power, and then rinsed in PBS, followed by incubation with 3% H_2_O_2_ and goat serum. They were stained with anti-PCNA (1:200), anti-E-cadherin (1:200), anti-ZO-1 (1:200), anti-AQP1 (1:250), anti-AQP2 (1:250), anti-NCC (1:250), anti-F4/80 (1:250), anti-TNF-α (1:250), anti-IL-1β (1:250), and anti-fibronectin (1:200) antibodies. Next day, they were washed and incubated with biotinylated goat anti-mouse/rabbit IgG for 1 h. The reaction results were visualized with DAB (1809270031, MXB-BIO, Fuzhou, China) and the slides were counterstained with hematoxylin.

### MitoSOX Fluorescence

Freshly kidneys were cut and 10-μm-thick slides were obtained to incubate in PBS containing 10 µM MitoSOX Red reagent (Invitrogen, M36008) for 40 min. Then the sections were rinsed in PBS and mounted onto microscope slides.

### Electron Microscopy

Renal cortex was fixed with 2.5% glutaraldehyde in 150 mM cacodylate solution, post-fixed with 1% osmium tetroxide. Subsequent ultrathin sections (50–80 nm) were contrasted by uranyl acetate and lead citrate, and observed with electron microscope (Hitachi H-7650, Japan).

### Western Blot

Kidney sample were lysed with the lysis buffer and protein concentration was measured with BCA Protein Assay Kit (Beyotime). Samples were separated by 10% SDS-PAGE, transferred to PVDF membranes and then blocked in 5% milk for 1 h. The primary antibodies were detected, including anti-HIF-1α (1:500), anti-E-cadherin (1:1,000), anti-ZO-1 (1:1,000), anti-AQP1 (1:1,000), anti-AQP2 (1:1,000), anti-Mfn1 (1:1,000), anti-Opa1(1:1,000), anti-Fis1 (1:1,000), anti-Drp1 (1:1,000), anti-TNF-α (1:1,500), anti-IL-1β (1:1,500), anti-vimentin (1:1,000), anti-α-SMA (1:1,000), and anti-β-actin (1:3,000) antibodies. Then the blots were incubated with the corresponding secondary antibodies (1:10,000, Dako).

### Quantitative Real-Time PCR

RNA extraction and real-time PCR were performed in accordance with the previous procedure (Zhang et al., 2017). In brief, RNA from kidneys was isolated using Trizol reagent (Vazyme) according to manufacturer^,^ skit, and then the RNA was reverse-transcribed to cDNA following the PrimeScript RT reagent kit. At last, amplification of RT-PCR was following the protocols with a SYBR Green Mix (Vazyme) (Li et al., 2020). The primers were as follows:

HIF-1α forward: 5′-TCATCGGAAACTCCAAAGCCA-3′and reverse:
5′-GGC​TGG​GAA​AAG​TTA​GGA​GTG-3′; collagen I forward:
5′-GGC​GGT​GCA​CAG​TCA​GAC​CAT -3′ and reverse:
5′-CCA​GTT​GGT​AAT​GCC​ATG​T-3′; Fn forward:
5′-ATG​TGG​ACC​CCT​CCT​GAT​AGT -3′ and reverse:
5′-GCC​CAG​TGA​TTT​CAG​CAA​AGG-3 ′; TNF-α forward:
5′-CCC​TCA​CAC​TCA​GAT​CAT​CTT​CT-3′ and reverse:
5′-GCT​ACG​ACG​TGG​GCT​ACA​G-3’; IL-6 forward:
5′-GAGGATACCACTCCCAACAGACC-3′and reverse:
5′-AAG​TGC​ATC​ATC​GTT​GTT​CAT​ACA-3’; α-SMA forward:
5′-CTTCGCTGGTGATGATGCTC-3′and reverse:
5′-GTT​GGT​GAT​GAT​GCC​GTG​TT-3’; β-actin forward:
5′-GGC​TGT​ATT​CCC​CTC​CAT​CG-3′ and reverse:
5′-CCA​GTT​GGT​AAT​GCC​ATG​T-3′;

The relative mRNA levels were analyzed using the 2^−ΔΔCt^ method.

### Statistical Analysis

The values presented were expressed as means ± standard deviations. Statistical comparisons were determined by one-way ANOVA test using SPSS software v.21.0, followed by the Bonferroni test. Values of *p* < 0.05 were considered statistically significant.

## Results

### FG-4592 Pretreatment Ameliorated FA-Induced Kidney Injury and Facilitated Tubular Recovery

**FIGURE 1:**
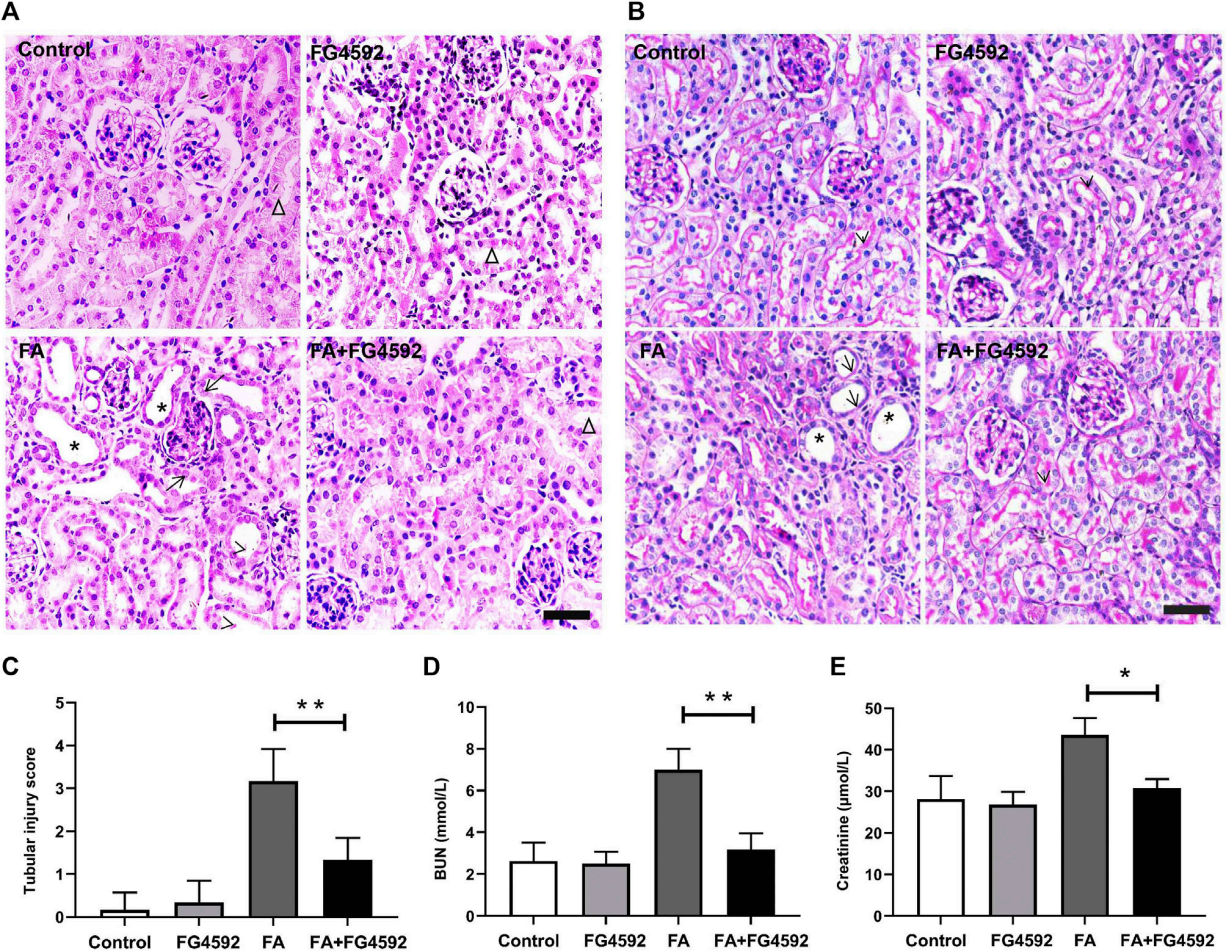
FG-4592 pretreatment ameliorated FA-induced renal damage and facilitated tubular recovery. **(A)** Renal histological assessment by HE staining. The asterisks indicate dilated tubules, the arrows indicate swollen epithelial cells, the arrowheads indicate tubules with cellular debris, the triangles indicate the normal tubule. **(B)** Renal histological assessment by PAS staining. The asterisks indicate tubular ectasia, the arrows indicate loss of tubular cells, and the arrowheads indicate the brush border. **(C)** Tissue injury scores relying on HE staining. **(D)** Serum BUN. **(E)** Serum creatinine. The data are expressed as the mean ± SE. Scale = 50 μm, *p < 0.05, **p < 0.01.

We conducted pathological staining to confirm whether FG-4592 pretreatment could alleviate kidney injury induced by FA overdose injection and promote tubular recovery on the 7th day. Histopathological examination of HE (Figure 1A) and PAS (Figure 1B) staining showed that FA induced severe lesions in the tubules with obvious maladaptive repair, including tubular dilation, tubular epithelial cell edema or loss, and inflammatory cell infiltration into the interstitium. In contrast, FG-4592 pretreatment dramatically alleviated the lesions and facilitated recovery, as indicated by an improved tubular structure and decreased tubular injury score (Figure 1C). Consistent with the histological changes, the FA group showed deteriorated renal function, as indicated by increased levels of BUN (Figure 1D) and creatinine (Figure 1E), but the levels were decreased with FG-4592 pretreatment.

### FG-4592 Pretreatment Promoted Structural Regeneration of Tubules on the 7th Day After FA Injection

**FIGURE 2:**
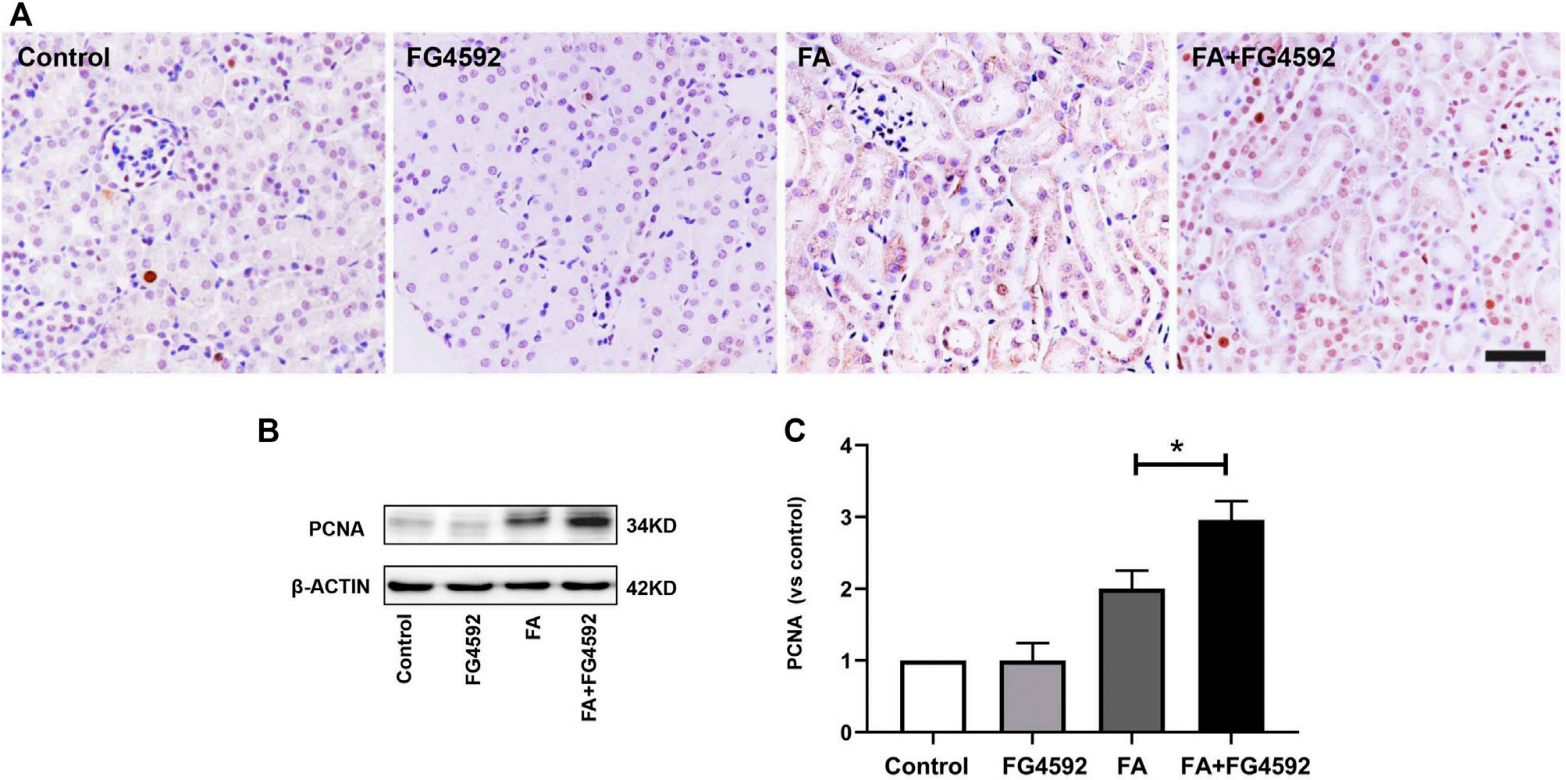
FG-4592 pretreatment promoted tubular cell proliferation in FA-induced renal damage. **(A)** Renal micrographs by IHC for PCNA. **(B)** Western blot analysis of PCNA in kidney lysates. **(C)** Relative abundance of PCNA. The data are expressed as the mean ± SE. Scale = 50 μm, *p < 0.05, **p < 0.01.

Regeneration of tubular epithelial cells can accelerate kidney repair after AKI (Jiang et al., 2015). To substantiate the regenerative effect, we examined tubular cell repopulation by staining for PCNA. As shown in Figure 2A, IHC analysis indicated that the number of PCNA-positive tubular cells was slightly increased on the 7th day in FA-injected mice compared with mice without FA administration; in contrast, the mice with FG-4592 pretreatment exhibited greater increases in the numbers of PCNA-positive tubular cells. Consistent with the results of IHC, Western blot analysis (Figures 2B,C) demonstrated that the level of PCNA was up-regulated after FA insult, and this up-regulation was further promoted with FG-4592 pretreatment, suggesting that FG-4592 pretreatment can facilitate tubular cell proliferation.

**FIGURE 3:**
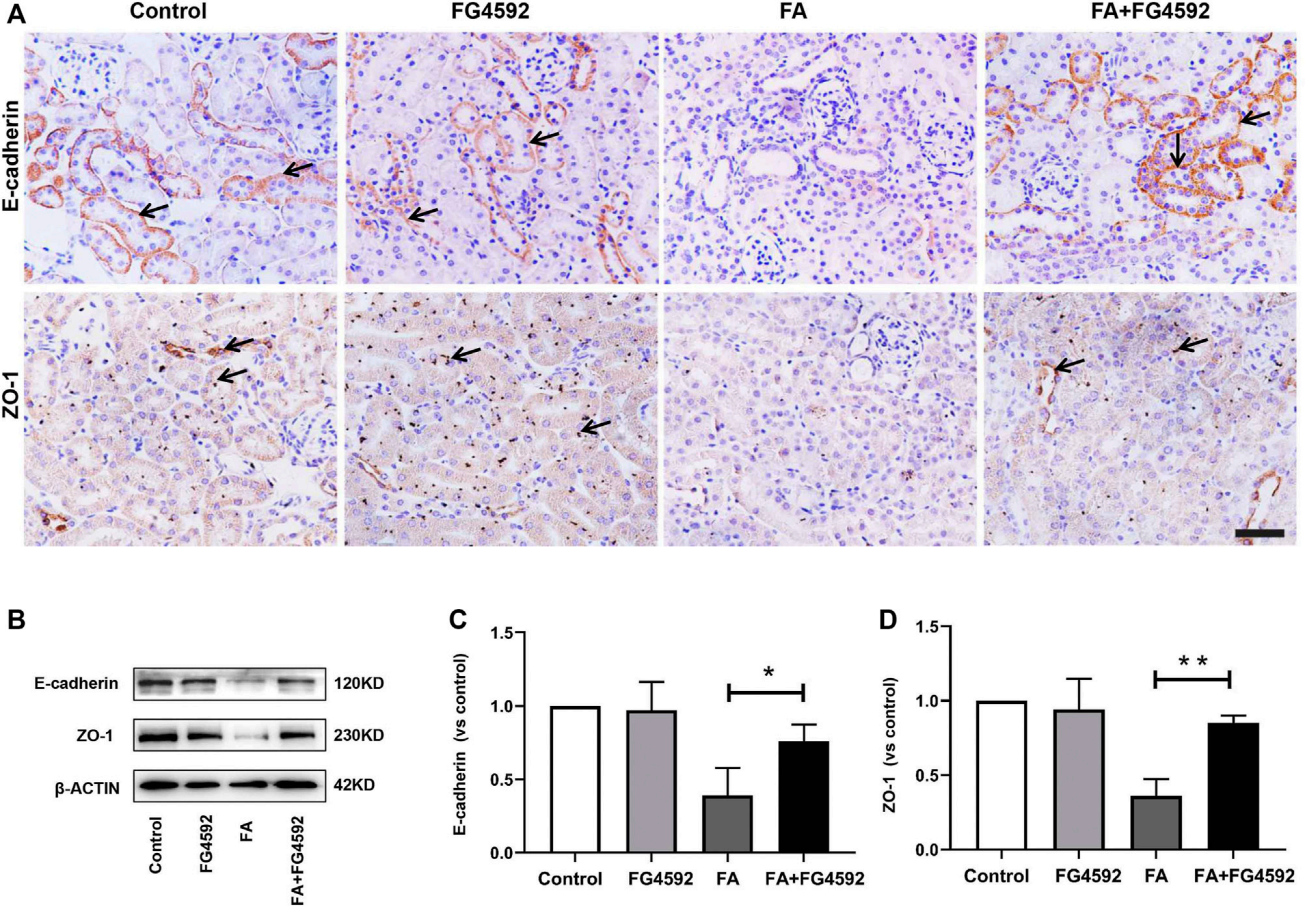
FG-4592 pretreatment promoted the restoration of tubular structural integrity in FA-induced renal damage. **(A)** Renal micrographs of IHC for E-cadherin and ZO-1. **(B)** Western blot analysis of E-cadherin and ZO-1. **(C)** Relative abundance of E-cadherin. **(D)** Relative abundance of ZO-1. The data are expressed as the mean ± SE. Scale = 50 μm, *p < 0.05, **p < 0.01.

In addition, recovery of the integrity of epithelial cell-cell junctions is essential for renal tubule repair. IHC analysis (Figure 3A) showed that E-cadherin, an adherens junction molecule, was highly expressed at the basolateral junction sites between neighboring cells in normal tubules. In comparison, the expression of E-cadherin was significantly reduced on the 7th day after FA injection but was restored with FG-4592 pretreatment. In addition, ZO-1 (a marker of tight junctions) was normally localized at the apical and basolateral junction sites between neighboring cells in the tubules but was significantly down-regulated after FA injection; however, its expression was partly restored with FG-4592 pretreatment. As described for IHC, these changes were further confirmed through Western blot analysis, and significantly decreased expression of E-cadherin and ZO-1 was found in FA-injured kidneys, whereas FG-4592 pretreatment reversed these effects, as depicted in Figures 3B–D.

### FG-4592 Pretreatment Facilitated Functional Recovery of Tubules on the 7th Day After FA Injection

**FIGURE 4:**
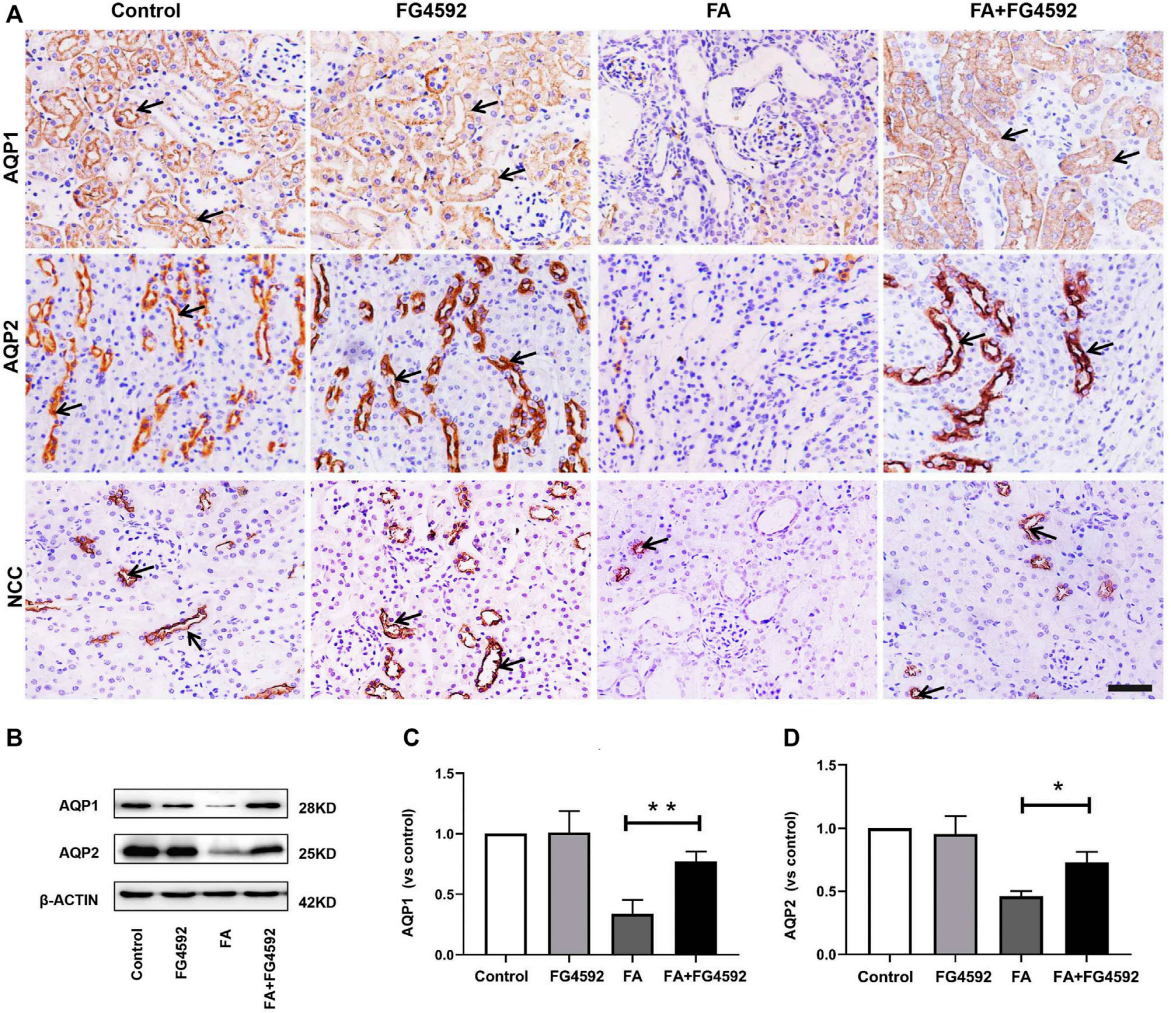
FG-4592 pretreatment promoted tubular functional recovery in FA-induced renal damage. **(A)** Renal micrographs of IHC for AQP1, AQP2, and NCC. **(B)** Western blot analysis of AQP1 and AQP2. **(C)** Relative abundance of AQP1. **(D)** Relative abundance of AQP2. The data are expressed as the mean ± SE. Scale = 50 μm, *p < 0.05, **p < 0.01.

The recovery of epithelial structural integrity plays a fundamental role in the restoration of tubular functions, such as absorption of fluid and solutes (Wang et al., 2008). We further detected functional recovery in different tubular segments with FG-4592 pretreatment on the 7th day after FA injection. IHC analysis (Figure 4A) demonstrated that AQP1 was widely expressed in the normal proximal tubules but down-regulated with dilation of the tubules after FA injection. However, FG-4592 pretreatment caused AQP1 to be re-expressed. Moreover, NCC, one of the essential sodium transporters, was highly expressed in normal distal tubules, but FA administration caused down-regulation of NCC. NCC expression was restored with FG-4592 pretreatment. Moreover, the level of AQP2 in the collecting ducts was significantly reduced after FA injection, while it was partially restored with FG-4592 pretreatment. Consistently, Western blot analysis indicated that compared with that in the FA group, the expression of AQP1 and AQP2 in the FA + FG-4592 group was significantly higher, as shown in Figures 4B–D.

### FG-4592 Pretreatment Alleviated Ferroptosis and Inflammation, and Further Inhibited Interstitial Fibrosis on the 7th Day After FA Injection

**FIGURE 5:**
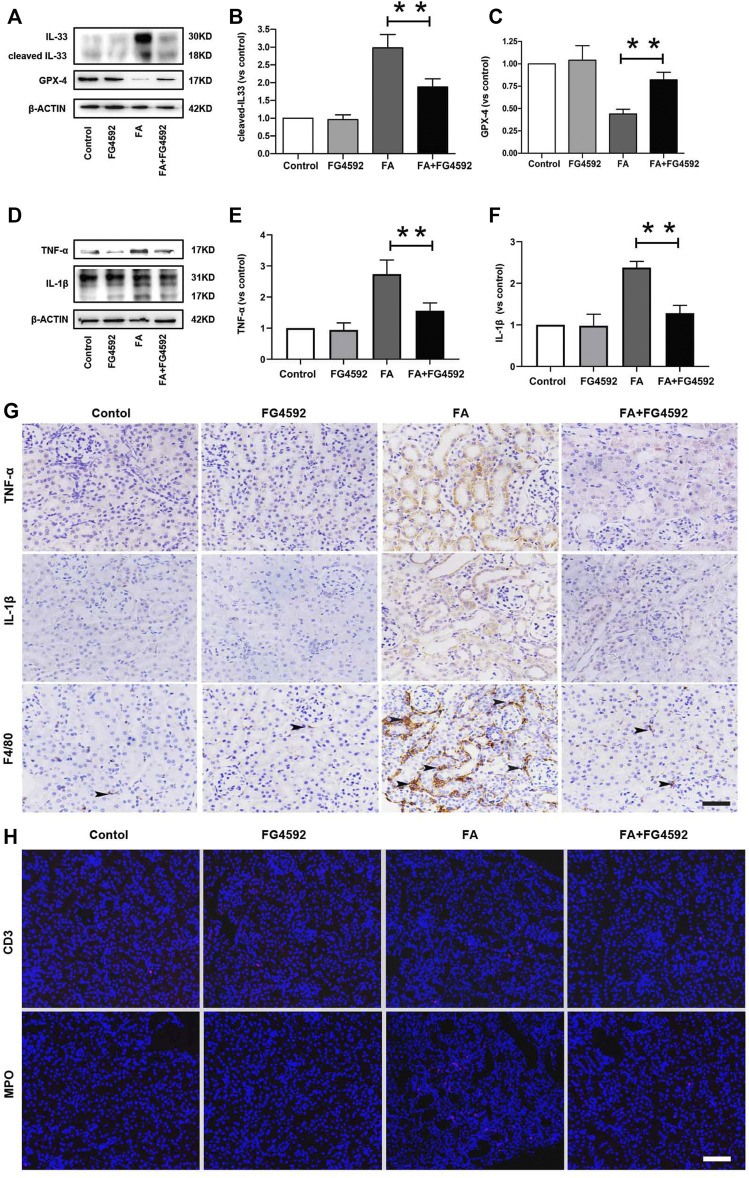
FG-4592 pretreatment alleviated ferroptosis and inflammation on the 7th day after FA injection. **(A)** Western blot analysis of IL-33 and GPX-4. **(B)** Relative abundance of cleaved-IL-33. **(C)** Relative abundance of GPX-4. **(D)** Western blot analysis of TNF-α and IL-1β. **(E)** Relative abundance of TNF-α. **(F)** Relative abundance of IL-1β. **(G)** Renal micrographs of IHC for TNF-α, IL-1β and F4/80-positive macrophages. **(H)** Renal micrographs of IF for CD3-positive lymphocytes and MPO-positive neutrophils. The data are expressed as the mean ± SE. Scale = 50 μm, *p < 0.05, **p < 0.01.

**FIGURE 6:**
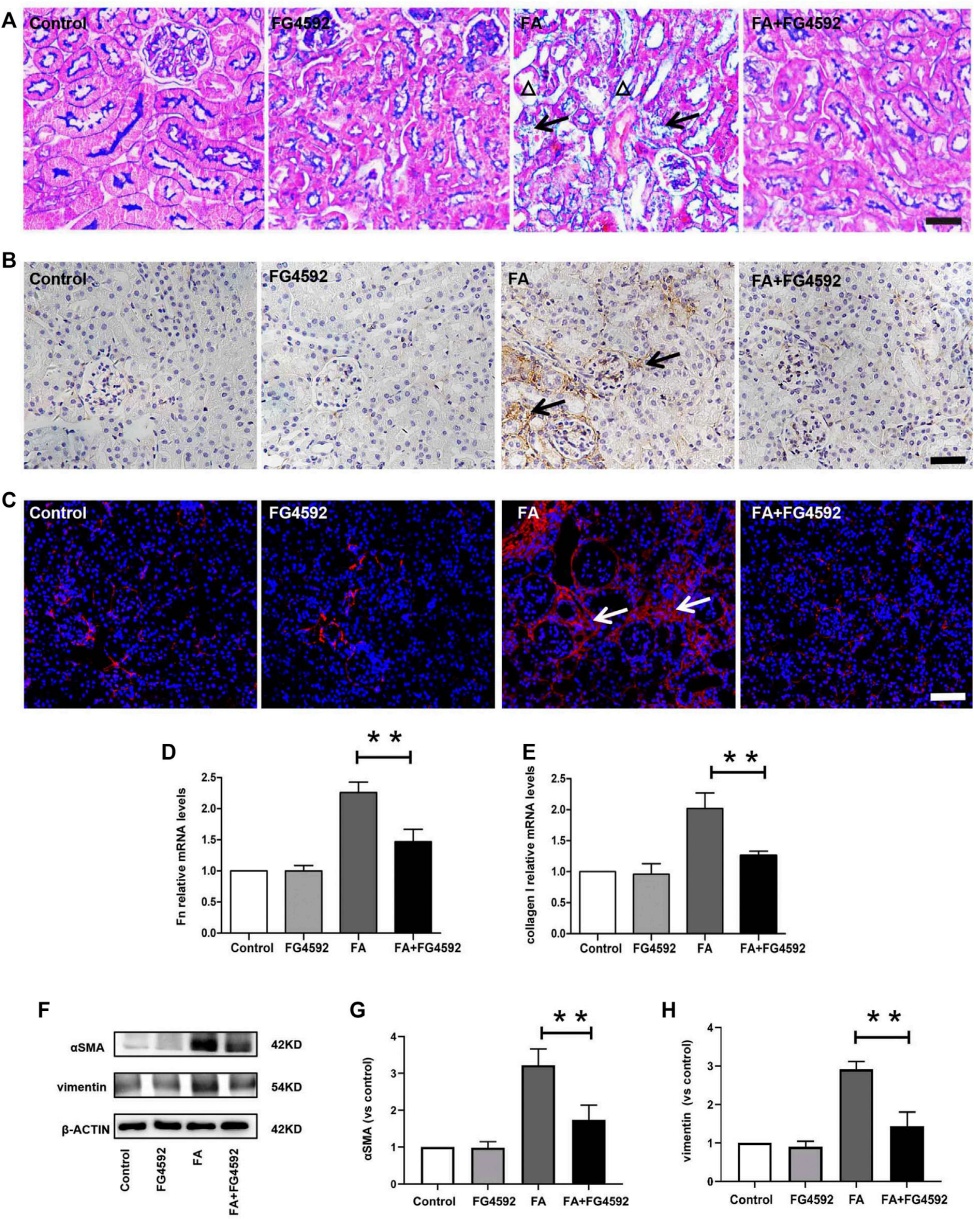
FG-4592 pretreatment inhibited renal fibrosis on the 7th day after FA injection. **(A)** Renal histological assessment by Masson trichrome staining. The triangles indicate the tubular atrophy and the arrows indicate collagen deposition. **(B)** Renal micrographs of IHC for Fibronectin. **(C)** Renal micrographs of IF for collagen I. **(D)** Relative mRNA level of Fibronectin. **(E)** Relative mRNA level of collagen I. **(F)** Western blot analysis of α-SMA and vimentin. **(G)** Relative abundance of α-SMA. **(H)** Relative abundance of vimentin. The data are expressed as the mean ± SE. Scale = 50 μm, *p < 0.05, **p < 0.01.

Ferroptosis was evidenced to be the major cause in FA-induced AKI, which has been reported to be a driver of other pathways of cell death (Martin-Sanchez et al., 2017) To better evaluate the effect of FG-4592 on the ferroptosis, the expressions of IL-33 and GPX4 were assessed by Western blot analysis. As depicted in Figures 5A–C, FA induced up-regulation of cleaved IL-33, while this effect could be partly inhibited with FG-4592 pretreatment. In addition, we observed that FG-4592 pretreatment was able to prevent the down-regulation of GPX4. Then we further explore whether FG-4592 pretreatment could inhibit inflammatory responses during the repair phase in FA-induced AKI. As described in Figures 5D–F, Western blot analysis indicated that FA induced up-regulation of the inflammatory cytokines TNF-α and IL-1β, which could be inhibited by FG-4592 pretreatment. Also, IHC analysis showed that TNF-α- and IL-1β-positive tubular cells were rarely expressed in non-FA-injected kidneys, but an abundance of these cells were observed after FA injection. However, these effects were prevented with FG-4592 pretreatment (Figure 5G). Furthermore, we assessed the levels of inflammatory cells, including macrophages, lymphocytes, and neutrophils on the 7th day after FA insult. As depicted in Figure 5G, few F4/80-positive cells were detectable in the kidneys of control or FG-4592-treated mice in kidney specimens by IHC staining. In contrast, following folic acid injury, abundant F4/80-positive cells were found in the renal interstitium, while pretreatment with FG-4592 markedly reduced the number of F4/80 + macrophages in the FA-injured kidneys. Consistently, FA-injured kidneys exhibited significant infiltrations of lymphocytes and neutrophils, as indicated by up-regulation of CD3- and MPO-positive cells, compared to that in the kidneys of control or FG-4592-treated mice, as shown by IF staining in Figure 5H, the numbers of these positive inflammatory cells in the kidneys were reduced by FG-4592 pretreatment, denoting anti-inflammatory activity. Furthermore, maladaptive repair after AKI aggravates inflammation, which may further drive CKD progression (Jiang et al., 2016a). To investigate the impact of FG-4592 pretreatment on interstitial fibrosis, Masson staining was performed. The results (Figure 6A) showed that FA injection induced severe tubular atrophy and collagen deposition in the interstitial space at Day 7 after FA injection, while FG-4592 pretreatment ameliorated these alterations. Moreover, we assessed some matrix proteins, such as collagen I and fibronectin. As depicted in Figures 6B,C, increased accumulation of fibronectin (Fn) and collagen I in the renal tubulointerstitium was observed in FA-injected mice at Day 7. The morphologic findings were further confirmed by RT-PCR, which showed that the gene levels of fibronectin and collagen I were upregulated after FA injection and were reduced by FG-4592 pretreatment (Figures 6D,E). In accordance with this findings, increased protein expression of α-SMA and vimentin in FA-injected kidneys was observed by Western blot analysis, while this effect was remarkably abrogated by FG-4592 pretreatment, indicating less epithelial-to-mesenchymal transition (EMT) and better recovery, as noted in Figures 6F–H.

### FG-4592 Pretreatment Suppressed the Activation of HIF-1α and Ameliorated Mitochondrial Dysfunction on the 7th Day After FA Injection

**FIGURE 7:**
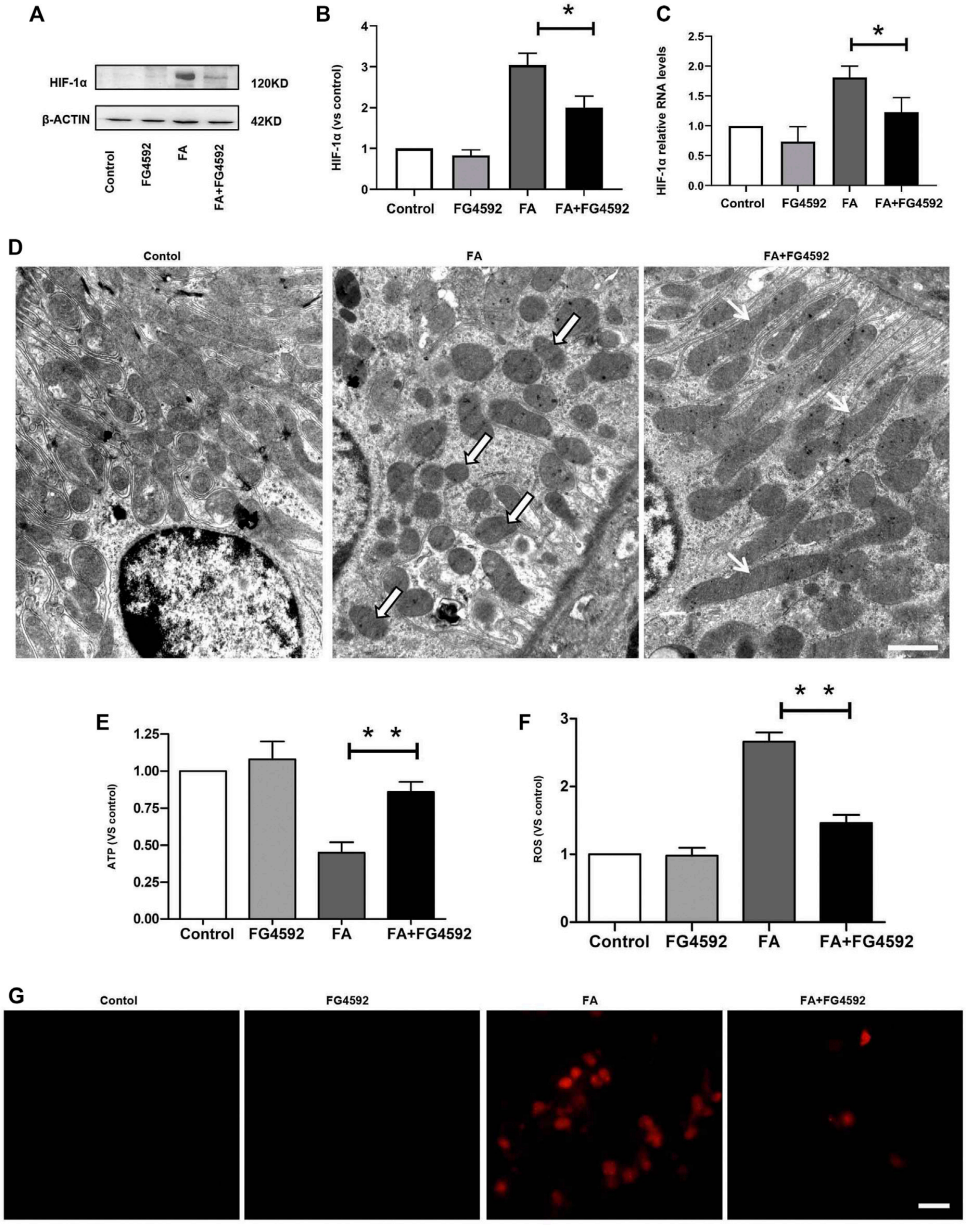
FG-4592 pretreatment suppressed HIF-1α activation and restored the alterations in mitochondrial morphology and function induced by FA injection. **(A)** Western blot analysis of HIF-1α. **(B)** Relative abundance of HIF-1α. **(C)** Relative mRNA level of HIF-1α. **(D)** Representative transmission electron microscopy for mitochondrial morphology at 20,000× magnification, Scale = 1 μm. Black and White Arrows indicate mitochondrial fragmentation; Black Arrows indicate elongated (>2 μm) mitochondria.). **(E)** ATP level in the kidney. **(F)** The level of ROS in the kidney. **(G)** Mitochondrial superoxide was detected by MitoSOX Red fluorogenic dye. The data are expressed as the mean ± SE. Scale = 50 μm, *p < 0.05, **p < 0.01.

To determine the role of FG-4592 pretreatment in the alterations in HIF-1α on the 7th day after FA administration, the protein and gene expression levels of HIF-1α were evaluated. Western blot analysis (Figures 7A,B) showed that the level of HIF-1α was increased after FA injection, whereas this increase was inhibited with FG-4592 pretreatment. A similar pattern was observed in the RT-PCR results (Figure 7C), in which FG-4592 pretreatment suppressed the up-regulation of HIF-1α mRNA levels induced by FA administration.

Furthermore, hypoxia-induced HIF-1α activation has been reported to be related to mitochondrial fragmentation (Zhang et al., 2018), which is one of the major factors that causes kidney injury and subsequent incomplete repair (Chung et al., 2019). Next, we assessed the ultrastructure of mitochondria (Figure 7D) and found that a large number of long filamentous mitochondria were present at the basolateral side in normal proximal tubular cells, while mitochondria in the perinuclear position showed cross-sectioning and appeared fragmented. In contrast, mitochondria were destroyed to a great extent and completely fragmented into swollen and short mitochondria with effacement of cristae after FA injection; moreover, this phenotype was accompanied by remarkably decreased ATP levels. While FG-4592 pretreatment could alleviate these alterations and promote ATP production, as shown in Figure 7E. In addition, mitochondrial dysfunction was characterized by excessive ROS production, which could be inhibited by FG-4592 pretreatment (Figure 7F). Moreover, the dynamic imbalance of mitochondria could induce increased level of mitochondrial ROS, which was further confirmed by analysis of the fluorescence intensity of MitoSOX, as shown in Figure 7G.

**FIGURE 8:**
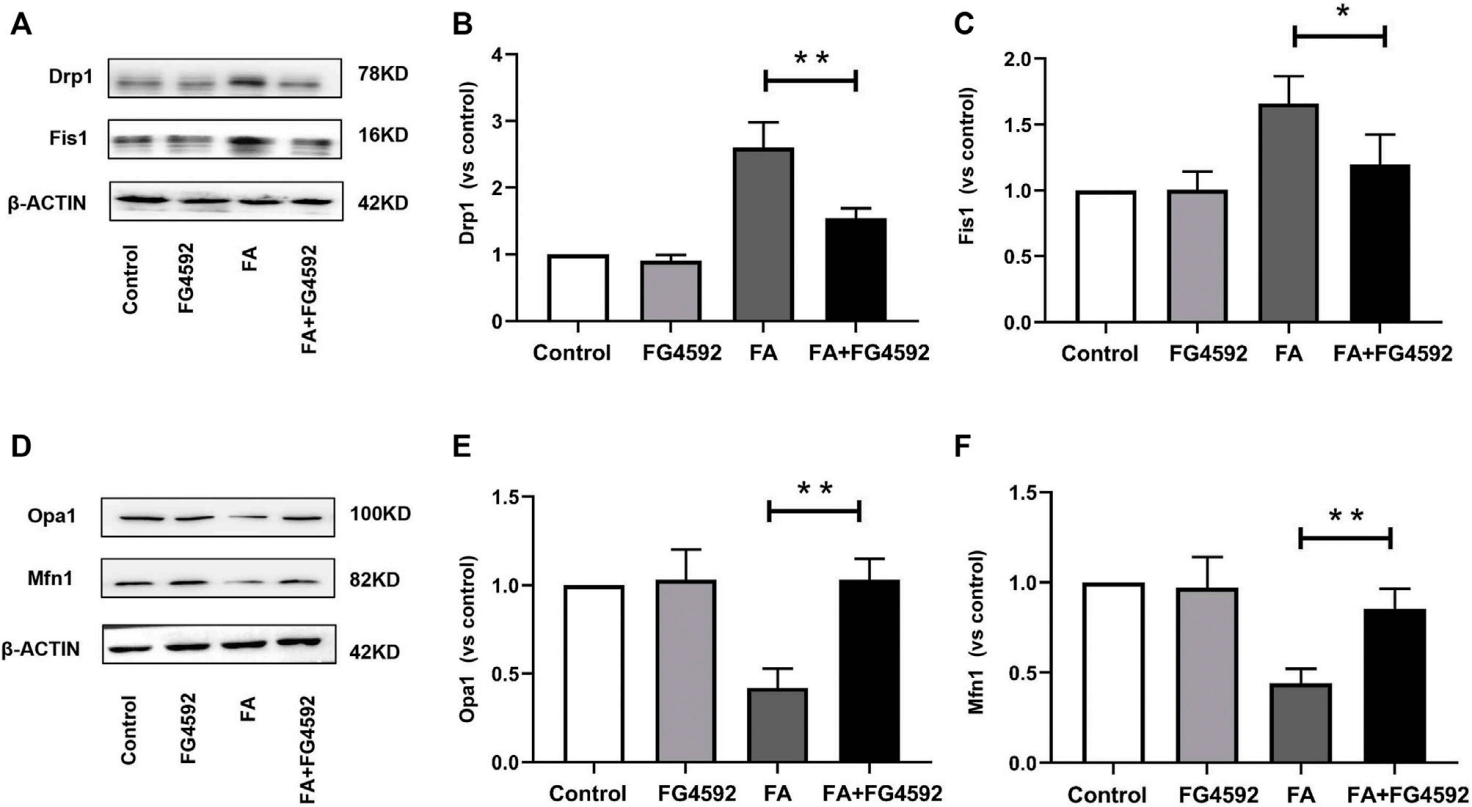
FG-4592 pretreatment attenuated FA-induced imbalance of mitochondrial dynamics. **(A)** Western blot analysis of Drp1 and Fis1. **(B)** Relative abundance of Drp1. **(C)** Relative abundance of Fis1. **(D)** Western blot analysis of Opa1 and Mfn1. **(E)** Relative abundance of Opa1. **(F)** Relative abundance of Mfn1. The data are expressed as the mean ± SE. Scale = 50 μm, *p < 0.05, **p < 0.01.

Moreover, we evaluated the effect of FG-4592 pretreatment on mitochondrial dynamics, which are mainly regulated by mitochondrial fusion and fission proteins. Western blot analysis showed increased expression of mitochondrial fission proteins (Fis1 and Drp1) (Figures 8A–C) and reduced expression of mitochondrial fusion proteins (Opa1 and Mfn1) (Figures 8D–F) after FA injection, indicating a shift in mitochondrial dynamics toward the fission process. On the other hand, FG-4592 pretreatment improved all these alterations, supporting the idea that FG-4592 pretreatment promoted the transition from mitochondrial fission to fusion. The dynamic alterations in mitochondrial activity indicated that mitochondrial damage induced by FA could be partially attenuated by FG-4592 pretreatment.

### FG-4592 Pretreatment Alleviated HK-2 Cells Injury Induced by TNF-α

**FIGURE 9:**
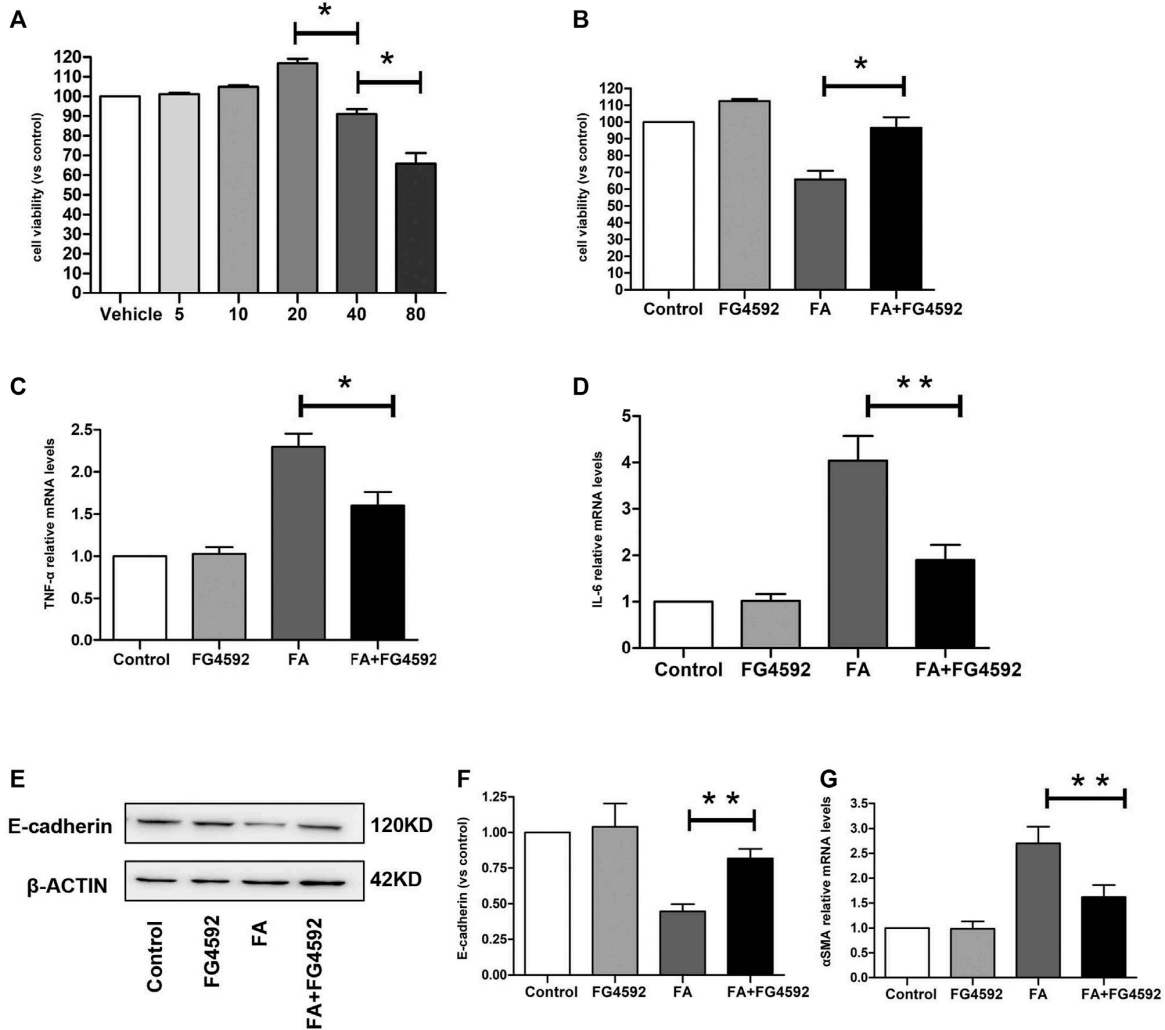
Effect of FG-4592 pretreatment on HK-2 cell induced by TNF-α. **(A)** CCK8 assay of the viability in HK-2 cells treated with FG-4592. **(B)** CCK8 assay of the viability in HK-2 cells treated by TNF-α with or without FG-4592 pretreatment. **(C)** Relative mRNA level of TNF-**α**. **(D)** Relative mRNA level of IL-6. **(E)** Western blot analysis of E-cadherin. **(F)** Relative abundance of E-cadherin. **(G)** Relative mRNA level of α-SMA. The data are expressed as the mean ± SE. *p < 0.05, **p < 0.01.

We evaluated the impact of FG-4592 on cell viability in the HK-2 cells at concentrations of 5–80 μM by CCK-8 assay. As shown in Figure 9A, FG-4592 had no cellular toxicity at concentrations of 5–20 μM, while FG-4592 at concentrations of 40 and 80 μM decreased cell viability. Moreover, we explored the effect of FG-4592 treatment on TNF-α-treated HK-2 cells and found that FG-4592 pretreatment could attenuate the reduced cell viability induced by TNF-α (Figure 9B). In addition, we measured the level of inflammation and found that FG-4592 at concentrations of 20 μM could down-regulate the gene levels of the inflammatory cytokines TNF-α and IL-6 induced by TNF-α (Figures 9C,D). In addition, we accessed the impact of FG-4592 on the expression of E-cadherin in HK-2 cells with or without FG-4592 pretreatment. As shown in Figures 9E,F, the Western blot results showed that E-cadherin was highly expressed in normal HK-2 cells but that the expression was significantly reduced after TNF-α stimulation, while the levels of E-cadherin were restored with FG-4592 pretreatment. Moreover, pretreatment with FG-4592 could markedly reduce the increased gene of α-SMA caused by TNF-α (Figure 9G, Supplementary Figure S1).

## Discussion

Mitochondrial dysfunction is a central factor to maladaptive repair after AKI (Stallons et al., 2014), and promoting the balance of mitochondrial dynamics is closely associated with the recovery of tubular cells that undergo sublethal injury (Wills et al., 2012). Thus, drugs that can ameliorate mitochondrial dysfunction may accelerate the recovery of renal tubules in AKI. Hypoxia and mitochondrial dynamics have been reported to be closely connected, while continuous hypoxia can induce the activation of HIF-1α, which negatively regulates mitochondrial function (LaGory et al., 2015; Jun et al., 2017).

FG-4592, a kind of prolyl hydroxylase inhibitor, has been used as a pretreatment to attenuate AKI through anti-oxidative stress in our previous study by increasing the levels of HIF-1α at the acute phase (Li et al., 2020); however, inhibition of HIF-1α mRNA was observed (data not shown) at the same time, which may be related to negative feedback. Here, we investigated the role of FG-4592 pretreatment in HIF-1α activity and renal damage in the repair phase. Tubular repair caused by FA overdose injection has been reported to commence from the 6th day, and maladaptive recovery occurs due to severe damage (Stallons et al., 2014). In view of this, we performed HE and PAS staining and observed the partial restoration of normal kidney architecture on the 7th day after FA administration, which was characterized by repopulation of some of the tubular epithelial cells. FG-4592 pretreatment facilitated recovery and alleviated tubular damage induced by FA. Furthermore, in contrast to the increases in HIF-1α protein levels observed on the 2nd day in FA-injected kidneys with FG-4592 pretreatment, inhibition of HIF-1α protein expression was observed on the 7th day. This was in line with the suppression of HIF-1α mRNA levels, indicating that the protein expression of HIF-1α is time-dependent with FG-4592 pretreatment and that the role of this protein may differ in different phases of AKI. In this regard, we speculate that FG-4592 pretreatment may be a powerful strategy to prevent the activation of HIF-1α induced by constant hypoxia during the recovery phase of AKI, thereby promoting tubular repair and improving its prognosis. Our study supports previous findings that suppression of HIF-1α activation induced by consistent hypoxia can alleviate tubular damage (Takiyama et al., 2011).

In addition, adaptive tubular repair is an important process to delay AKI progression, and tubular cell regeneration is a key event (Basile et al., 2016). It has been reported that fully differentiated tubular cells have equal chances of proliferation after injury (Humphreys et al., 2016; Soofi et al., 2020). In our study, increased cell proliferation was observed in the FG-4592-treated mice, indicating the stimulation of tubular repair. Furthermore, tubular cell proliferation drives restoration of tubular epithelial integrity, which is critical to the repair of AKI (Nony and Schnellmann 2003). E-cadherin, a major component of cell-cell AJs, plays an important role in tubular integrity and polarity (Gao et al., 2018). It has been demonstrated that the E-cadherin levels in tubular cells are significantly reduced in response to cisplatin, but tubular damage can be alleviated with restoration of E-cadherin expression (Ni et al., 2019). Another study has demonstrated that decreased expression of E-cadherin is part of the phenotype of epithelial-to-mesenchymal transition and is closely associated with renal fibrosis (Fragiadaki and Mason 2011). Moreover, the assembly of AJs is associated with that of TJs, and destruction of E-cadherin-mediated AJs also delays TJ assembly (Capaldo and Macara 2007). ZO-1, the critical TJ protein, is essential for tubular barrier formation. Eadon and colleagues observed decreased expression of ZO-1 in LPS-mediated AKI (Eadon et al., 2012). Our findings are consistent with these observations that FA injury induces disruption of tubular AJs and TJs, as reflected by down-regulation of E-cadherin and ZO-1 levels. However, FG-4592 pretreatment retained the expression of these molecules in tubular cells, indicating the ability to maintain the structural integrity of tubular cells. Along with the protective effects shown *in vivo*, FG-4592 pretreatment could also protect renal tubular cells against TNF-α induced down-regulation of E-cadherin *in vitro*.

Moreover, restoration of the tubular epithelial barrier is indispensable for maintaining tubular function and is mainly involved in reabsorption capacity and ion transport, which contribute to body fluid volume regulation and electrolyte handling (Pozzi and Zent 2010; Fattah and Vallon 2018). AQPs, especially AQP1 and AQP2, account for the regulation of water transport and urinary concentration (Agre et al., 2002). Previous studies have shown that AQP1, which is widely expressed in the proximal tubules as well as descending thin limbs, is downregulated by I/R injury, which contributes to urinary concentration defects (Ma et al., 1998). Moreover, endotoxemia can induce more severe tubular injury in AQP1-null mice than in wild-type mice, which is characterized by polyuria (Hua et al., 2019). In contrast, AQP1 overexpression can alleviate aristolochic acid-induced nephropathy (Anger et al., 2020). In addition, it has been reported that AQP1 levels in the proximal tubule together with AQP2 levels in the collecting duct are decreased in I/R-induced kidney injury, which results in impaired urinary osmolality (Kwon et al., 1998; Kortenoeven and Fenton 2014). Down-regulation of the sodium cotransporter, NCC, is also associated with dysfunction of sodium excretion and impaired urinary concentration ability in AKI (Bae et al., 2008; Yang et al., 2013). Furthermore, restoration of urinary concentration capacity may inhibit the transition from AKI to CKD (Asvapromtada et al., 2018). Our observations are in line with these reports. Specifically, we found that FA injury decreased the expression of AQP1, NCC, and AQP2 but that the expression of these molecules was restored with FG-4592 pretreatment, facilitating tubular functional recovery.

In addition, AKI is often accompanied by tubular epithelial necrosis, which could further inhibit the repair of injured kidneys. While ferroptosis was reported to act as an important mechanism that contributes to FA-induced acute renal damage, the protective effect of FA-induced AKI might be achieved by inhibiting ferroptosis (Hu et al., 2019). Similarly, we observed that FG-4592 pretreatment reversed the elevation in IL-33 and decrease in GPX4 induced by FA injection, suggesting that anti-ferroptosis may be the main pathway involved in the protection against FA-induced AKI. Moreover, maladaptive repair of tubules can further activate proinflammatory and profibrotic effects (Jiang et al., 2016b). Disruption of E-cadherin has been shown to promote inflammation in cisplatin-induced tubular injury (Gao et al., 2018). Restoration of AQP1 has been reported alleviate inflammation in LPS-induced renal damage (Li et al., 2019). Consistently, our discoveries indicate that FG-4592 pretreatment can reduce the recruitment of numerous macrophages, lymphocytes, and neutrophils, as well as the release of the inflammatory factors TNF-α and IL-1β induced by FA insult, thereby reducing renal damage of function and structure. Although we did not differentiate the subtypes of macrophages, a previous study has indicated that the majority of macrophages that infiltrate the interstitium on the 7th day after FA injection are M1 macrophages, which exhibit proinflammatory activities (Jiang et al., 2016b). In consistency, we observed that inflammatory factors were increased by stimulating HK-2 cells with TNF-α, while FG-4592 pretreatment could attenuate these alterations. Meanwhile, FG-4592 pretreatment could reduce α-SMA level, indicating the amelioration of EMT *in vitro*. What is more, down-regulation of HIF-1α along with mitochondrial stress modulation strategy was demonstrated to curtail inflammation in mammary gland chemoprevention (Roy et al., 2017).

Mitochondria are the main energy sources in tubular cells, but mitochondrial dysfunction can lead to maladaptive progression of AKI and further accelerate fibrosis development (Yu and Bonventre 2020). It has been reported that mitochondrial fragmentation is significantly increased in tubular epithelial cells treated with cisplatin or glycerol (Brooks et al., 2009). Moreover, maladaptive repair from FA-induced AKI is recognized as a complex multifactorial process in which mitochondrial impairments play important roles (Sun et al., 2019). Through subcellular structure analysis, we found that mitochondrial fragmentation or mitochondrial shrinkage induced by FA injection increased under hypoxia, leading to a reduction in intracellular ATP, while FG-4592 pretreatment improved the situation. Moreover, mitochondrial damage not only contributes to decreases in ATP levels but also results in excessive production of ROS, which adversely affects cell survival and recovery (Apostolova and Victor 2015). Consistently, a marked elevation in mitochondrial ROS after FA injection was observed in our study, and this elevation was attenuated by FG-4592 pretreatment. In addition, up-regulation of HIF-1α level was found to be closely associated with dysfunction of mitochondria, which can induce mammary gland carcinoma progression (Singh et al., 2021b). Polyl hydroxylase (PHD-2) activation was considered as a novel strategy to control HIF-1α to modulate mitochondrial stress in mammary gland pathophysiology of ER + subtype (Singh et al., 2021a). Meanwhile, down-regulation of HIF-1α/fatty acid synthase co-axis was shown to combat tumor growth in mammary gland carcinoma by activating PHD-2 (Gautam et al., 2018) (Manral et al., 2016).

Furthermore, inactivation of HIF-1α induced by consistent hypoxia can ameliorate mitochondrial dysfunction and alleviate tubular damage (Takiyama et al., 2011). During AKI, mitochondria experience dynamic imbalance, which depletes cellular ATP and further induces the loss of tight junctions and adherens junctions (Molitoris 2004). The balance of mitochondrial fusion and fission is important for appropriate mitochondrial morphology and function to meet the energy needs of tubular cells and restore ATP production. Targeted treatment with drugs that ameliorate mitochondrial function by inhibiting the expression of Drp1 and restoring the expression of Mfn2 have been reported to ameliorate AKI (Ishimoto and Inagi 2016). Drp1, a GTPase, regulates mitochondrial fission processes, but its upregulation is detrimental because it enhances mitochondrial fragmentation (Tang et al., 2013). Perry et al. showed that suppression of Drp1 expression in proximal tubular cells accelerated recovery, therefore attenuating fibrosis following renal IR (Perry et al., 2018). Consistent with these mechanisms, we found that FA induced mitochondrial fragmentation through increased expression of fission proteins (Drp1 and Fis1) and decreased expression of fusion proteins (Opa1 and Mfn1). In contrast, FG-4592 pretreatment recovered the balance of mitochondrial dynamics by reversing these changes, therefore promoting mitochondrial elongation. Moreover, mitochondrial hyperfission is critical for promoting cellular necrosis, which is characterized by a reduced number of mitochondrial cristae and the change of mitochondrial membrane potential, resulting in cell volume shrinkage (Spurlock et al., 2020). Specifically, GPX4 is partially located in the intermembrane spaces of mitochondria to maintain mitochondrial membrane potential, which can counteract ferroptotic cell death. GPX4-ablated cells contain swollen mitochondria with disappearance of cristae and a lamellar architecture (Baseler et al., 2013).

In summary, our findings indicate that mitochondrial dysfunction may be pathogenic in the maladaptive repair of FA-induced AKI. Moreover, we show the beneficial effects of FG-4592 pretreatment against renal damage associated with FA overdose injection. FG-4592 decreases tubular structural and functional injury, inhibits tubular ferroptosis and inflammation, and specifically accelerates recovery from tubular injury. As a result of these actions, FG-4592 pretreatment decreases interstitial fibrosis. Finally, we identified facilitation of the homeostasis of mitochondrial dynamics as a key pathway involved in the protective effects of FG-4592 pretreatment. FG-4592 improves the balance of mitochondrial dynamics by inhibiting mitochondrial fission and promoting mitochondrial fusion. The mechanism underlying this phenomenon involves downregulation of the primary mediator proteins of mitochondrial fission (Drp1 and Fis1) and upregulation of fusion proteins (Opa1 and Mfn1). Overall, the major consequence of FG-4592-mediated balancing of mitochondrial dynamics is recovery of functional mitochondria; therefore, FG-4592 is a potential therapeutic agent for nephrotoxic AKI.

## Data Availability

The original contributions presented in the study are included in the article/Supplementary Material, further inquiries can be directed to the corresponding author.
