# Supplementary figures and images for: Roxadustat (FG-4592) Facilitates Recovery From Renal Damage by Ameliorating Mitochondrial Dysfunction Induced by Folic Acid
Source: Front Pharmacol. 2022 Feb 25;12:788977. doi: 10.3389/fphar.2021.788977 (PMC8915431; doi:10.3389/fphar.2021.788977)

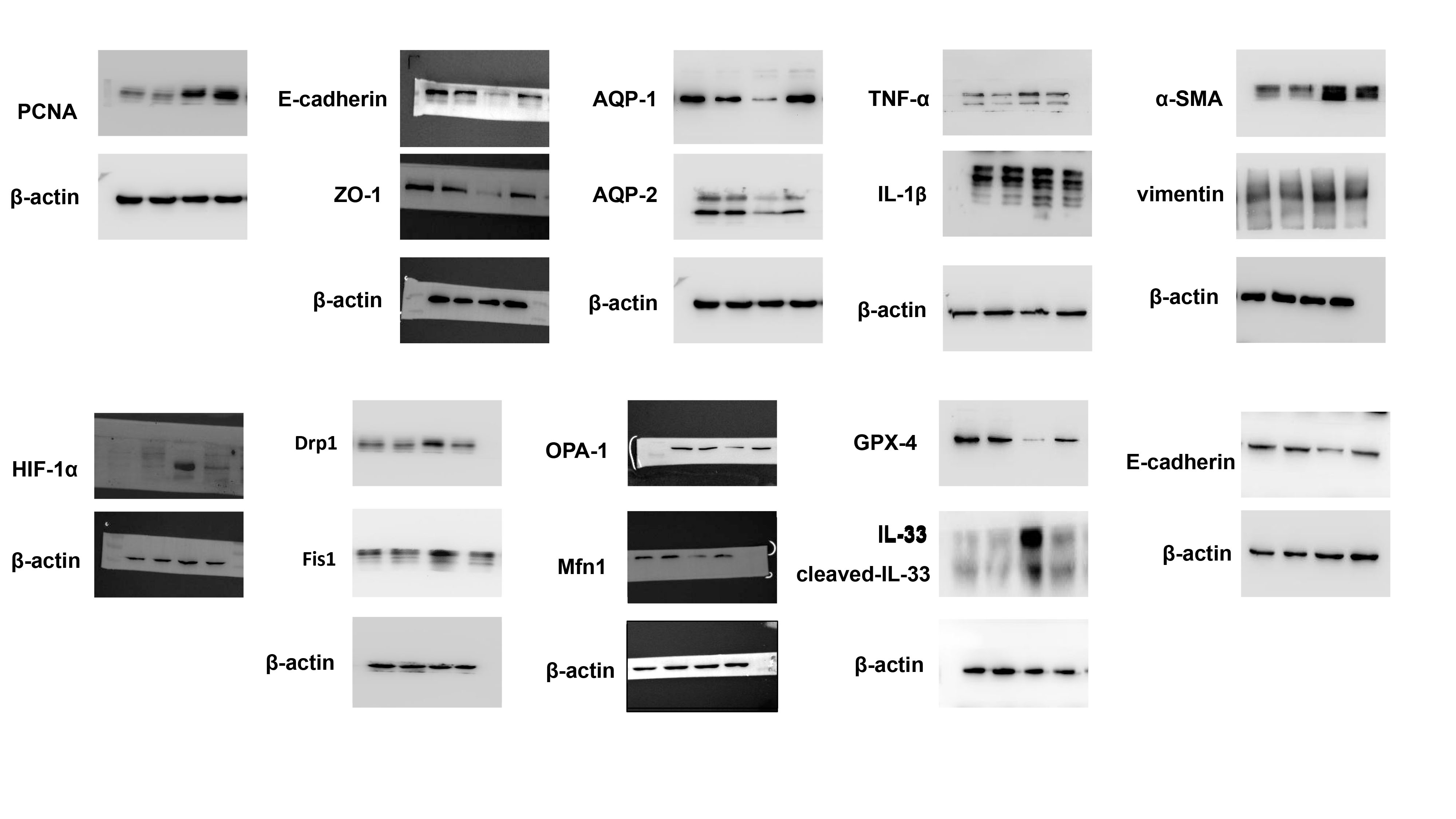

Supplement: Supplementary file 1 [file Image1.jpg]
